# Transposon-mediated Chromosomal Integration of Transgenes in the Parasitic Nematode *Strongyloides ratti* and Establishment of Stable Transgenic Lines

**DOI:** 10.1371/journal.ppat.1002871

**Published:** 2012-08-09

**Authors:** Hongguang Shao, Xinshe Li, Thomas J. Nolan, Holman C. Massey, Edward J. Pearce, James B. Lok

**Affiliations:** 1 Department of Pathobiology, School of Veterinary Medicine, University of Pennsylvania, Philadelphia, Pennsylvania, United States of America; 2 Department of Pathology and Immunology, School of Medicine, Washington University, St. Louis, Missouri, United States of America; George Washington University Medical Center, United States of America

## Abstract

Genetic transformation is a potential tool for analyzing gene function and thereby identifying new drug and vaccine targets in parasitic nematodes, which adversely affect more than one billion people. We have previously developed a robust system for transgenesis in *Strongyloides* spp. using gonadal microinjection for gene transfer. In this system, transgenes are expressed in promoter-regulated fashion in the F1 but are silenced in subsequent generations, presumably because of their location in repetitive episomal arrays. To counteract this silencing, we explored transposon-mediated chromosomal integration of transgenes in *S. ratti*. To this end, we constructed a donor vector encoding green fluorescent protein (GFP) under the control of the *Ss-act*-2 promoter with flanking inverted tandem repeats specific for the *piggyBac* transposon. In three experiments, free-living *Strongyloides ratti* females were transformed with this donor vector and a helper plasmid encoding the *piggyBac* transposase. A mean of 7.9% of F1 larvae were GFP-positive. We inoculated rats with GFP-positive F1 infective larvae, and 0.5% of 6014 F2 individuals resulting from this host passage were GFP-positive. We cultured GFP-positive F2 individuals to produce GFP-positive F3 L3i for additional rounds of host and culture passage. Mean GFP expression frequencies in subsequent generations were 15.6% in the F3, 99.0% in the F4, 82.4% in the F5 and 98.7% in the F6. The resulting transgenic lines now have virtually uniform GFP expression among all progeny after at least 10 generations of passage. Chromosomal integration of the reporter transgenes was confirmed by Southern blotting and splinkerette PCR, which revealed the transgene flanked by *S. ratti* genomic sequences corresponding to five discrete integration sites. BLAST searches of flanking sequences against the *S. ratti* genome revealed integrations in five contigs. This result provides the basis for two powerful functional genomic tools in *S. ratti*: heritable transgenesis and insertional mutagenesis.

## Author Summary

Parasitic roundworms sicken and debilitate over one billion people, most of whom subsist on less than two US dollars per day. There are no vaccines and few drugs available to treat and prevent these infections. Basic research leading to new therapies has been hampered because we lack methods to study gene function in parasitic roundworms. One such method is transgenesis, a process by which gene function is inferred by studying the effects of transferring native or altered copies of genes into subject organisms. Our laboratory has developed a system for transferring synthetic genes into parasitic roundworms of the genus *Strongyloides* and for obtaining temporary expression of these “transgenes”. Until now, however, we have been unable to propagate these transgenic parasites in the laboratory. This paper describes a new technique that allows us to establish and maintain self-perpetuating lines of transgenic parasites for study. This represents a fundamental advance in the methodology for studying gene function in parasitic roundworms and should greatly facilitate the discovery of new therapies.

## Introduction

Parasitic nematodes have an enormous impact on human welfare, infecting over a billion people and causing debilitating, disfiguring or blinding disease in hundreds of millions [1], [2], [3]. More insidious effects of these parasites include complications in pregnancy and physical and cognitive deficits in children [4], [5]. Parasitic nematodes severely degrade the health of domestic animals, bringing about significant economic losses in developed agricultural production systems [6], [7] and heighten food insecurity in marginal economies [8].

The need for ongoing research into new agents to prevent or treat parasitic nematode infection is acute. There are currently no effective vaccines available for these parasitisms, and chemotherapy is based on a relatively small arsenal of anthelmintic drugs [9], [10]. Resistance to each of these is widespread among parasites of livestock [11], [12], and suboptimal anthelmintic treatment responses in human clinical settings may signal genetically based resistance arising in populations of nematode parasites of humans [13], [14], [15], [16]. Screening has and will likely continue to identify new candidate anthelmintics against nematodes. Increasingly, however, alternative approaches involving rational design of agents directed at defined molecular targets are important in developing new drugs and identifying potential vaccine candidates in other infectious disease systems and have recently been brought to bear on parasitic nematodes [17]. Burgeoning descriptive genomic and transcriptomic resources for parasitic nematodes notwithstanding [18], [19], [20], [21], [22], [23], functional characterization and validation of such molecular targets in these worms has been hampered by the lack of robust functional genomic tools such as transgenesis and targeted gene silencing or disruption. For this reason our laboratory has worked towards a system for transgenesis in parasitic nematodes of the genus *Strongyloides* [24], [25], [26], [27].

We focus on *Strongyloides* spp. because unlike most parasitic nematodes, which display an invariant pattern of development to infective larvae outside the host, these worms execute one or more generations of free-living development between parasitic generations [28]. The free-living females of *Strongyloides* constitute an advantageous point of attack for gene transfer because they may be cultured in vitro and because morphological similarities between them and hermaphrodites of the free-living nematode *Caenorhabditis elegans* have made it relatively straightforward to adapt the technique of gonadal microinjection, originally devised for transgenesis in *C. elegans* [29], [30], [31], to these parasites [27]. This technique, along with the design of vector constructs containing both 5′ and 3′ regulatory sequences from *Strongyloides* sp. has enabled a robust system for generating transgene expressing *S. stercoralis* larvae of the F1 generation following gene transfer [24], [25]. In an effort to establish stable transgenic lines, F1 transgenic larvae of *S. stercoralis* have been reared to infective third-stages and used to establish patent infections in gerbils [24], [25]. Transgene expression has been observed in parasitic females recovered from the intestines of these animals. Recently we demonstrated that transgene constructs containing regulatory sequences from *S. stercoralis* are expressed at roughly equal frequencies and in virtually identical anatomical patterns in *Strongyloides ratti* [26]. When F1 transgenics of *S. stercoralis* [24], [25] and *S. ratti* (unpublished) transformed with conventional plasmid vectors are subjected to host passage, a substantial proportion of their F2 progeny harbor transgene sequences, but these are not expressed in this or subsequent generations of passage.

We assume that like *C. elegans*, *Strongyloides* spp. assemble the majority of microinjected transgenes into highly repetitive episomal arrays [30], [31], and we hypothesize that *Strongyloides* spp. actively silence these because of their episomal location, their highly repetitive character [32] or both. We have addressed both of these scenarios by attempting low-copy integration of transgenes into the chromosomes of *S. ratti* using the *piggyBac* transposon system [33]. As an additional precaution against epigenetic silencing, we assessed the ability of the gypsy retroviral insulator sequences from *Drosophila* [34] to sustain transgene expression during host passage. Preliminary experiments with this system yielded the first transgene expressing individuals of the F2 generation in *S. stercoralis* and *S. ratti* observed to date [33]. In the present study we confirmed this finding in *S. ratti*, demonstrated that the *piggyBac* system results in chromosomal integration of transgenes, and we established lines of this parasite that stably transmit and express integrated transgenes in virtually all progeny.

## Results/Discussion

### Constructs incorporating regulatory elements of the *piggyBac* transposon give reporter gene expression in F2 transgenic larvae of *S. ratti*

**Figure 1:**
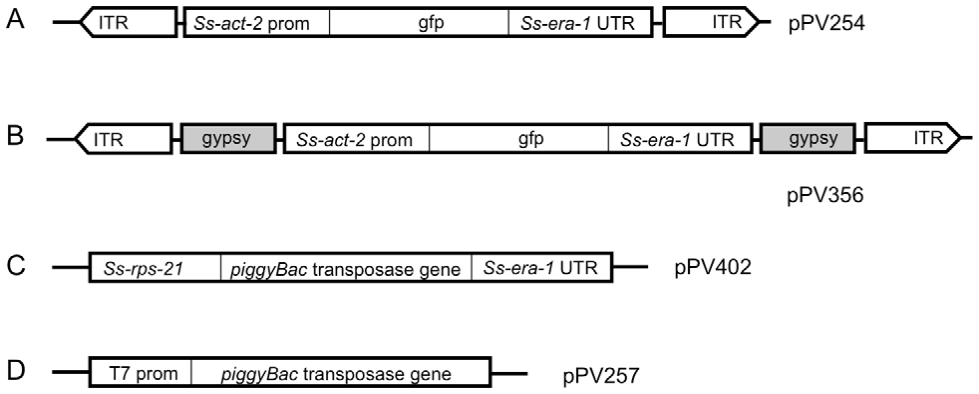
Vector constructs used to establish stable transgenic lines of *Strongyloides ratti* incorporate elements of the *piggyBac* transposon system. **A**) Donor vector pPV254 incorporating a fluorescent reporter gene in which expression of GFP is driven by the *Ss-act-2* promoter and terminated by the *Ss era-1* 3′ UTR. The reporter transgene in pPV254 is flanked by the inverted terminal repeats (ITR) plus internal sequences common to *piggyBac* transposable elements. **B**) Donor vector pPV356, which is like pPV254 in all respects except that the coding sequence is flanked by the gypsy retroviral insulator sequences from *Drosophila*. **C**) Helper vector pPV402 in which expression of the *piggyBac* transposase gene is driven by *Ss-rps-21* promoter and terminated by the *Ss-era-1* 3′ UTR. **D**) Plasmid pPV257 for in vitro transcription of mRNA encoding the *piggyBac* transposase under the T7 promoter. In lieu of helper vector pPV402, this mRNA was capped, tailed and co-injected with donor vector pPV356 in Experiment 2.

To achieve low-copy integration of transgenes in *Strongyloides* we designed constructs that incorporate regulatory elements of the *piggyBac* transposon system (Fig. 1). The transgene encoded in both the donor constructs tested (Fig. 1A, B) included a previously reported GFP expression cassette in which expression is directed to the body wall by the promoter for the cellular actin gene *Ss-act-2* [24]. In both donor constructs, this reporter transgene was flanked by the inverted terminal repeats specific for the *piggyBac* transposon [35] as well as internal transposon sequences shown to be necessary for efficient transposition in *Drosophila* [36]. In construct pPV356 (Fig. 1B), the reporter transgene is also flanked by the gypsy retroviral insulator sequences as a further precaution against epigenetic silencing. It is worth noting that in an effort to define a minimal functional transposon for studies of gene function in insects [36], the internal transposon sequences in the *piggyBac* encoding vector pXLBacII, from which we derived the *piggyBac* elements for our vectors, have been significantly reduced in length compared to the naturally occurring transposon from *Trichoplusia ni* [37]. Although this minimal transposon worked under the conditions obtaining in this initial study with *S. ratti* and mobilizes efficiently in both *Trichoplusia* [36] and *Drosophila* [38], it is possible that its efficiency could be increased in future studies of *Strongyloides* and other parasites by optimizing the lengths of internal sequences in the donor vector. As discussed below, this issue may be particularly important in attempts to establish stable lines of dioecious parasitic nematodes with integrated transgenes. Function of the *piggyBac* transposase was encoded either in a second plasmid vector, the helper (pPV402, Fig. 1C), in which the enzyme was expressed ubiquitously [24] under the promoter for the ribosomal small subunit gene *Ss-rps-21*, or in capped and tailed synthetic mRNA transcribed in vitro under the T7 promoter from linearized plasmid pPV257 (Fig. 1D).

In preliminary experiments [33], transforming *S. stercoralis* and *S. ratti* with pPV356 along with mRNA encoding the *piggyBac* transposase resulted in efficient production of F1 transgenics in both parasites, and, following passage of these through gerbils or rats, respectively, the first transgene-expressing F2 parasites observed to date. Given the very low numbers of F2 transgenic *S. stercoralis* produced relative to the approximately 50 third-stage larvae required to establish a patent infection in the gerbil or dog, we focused the present study on *S. ratti*. Transgenes with regulatory elements from *S. stercoralis* are expressed in virtually identical patterns and at equal frequencies in *S. ratti* [26]. Most importantly, the availability of a well-adapted laboratory host, the rat, makes it possible to establish patent infections with as few as one or two infective larvae [26], [33].

Transformation of *S. ratti* with pPV356 in the absence of transposase-encoding helper constructs gave efficient transmission and expression of transgenes in F1 progeny, but passage of these through rats failed to yield any transgene-expressing larvae in the F2 generation (Table 1, Experiment 1). Transformation of *S. ratti* with donor plasmid pPV356 combined with capped RNA encoding the *piggyBac* transposase gave efficient transgene transmission and expression in F1 larvae, and it resulted in a small proportion of F2 individuals expressing the transgene (Table 1, Experiment 2). This finding is consistent with our hypothesis that silencing observed in *Strongyloides* spp. transformed with plasmid vectors stems from the assembly of the encoded transgenes into episomal arrays and that this silencing can be circumvented by integration of the transgenes into the chromosomes of the parasites. Replicate experiments (Experiments 3 and 4, Table 1), in which worms were transformed with pPV356 and the helper plasmid pPV402, yielded markedly higher proportions of F2 parasites expressing the reporter transgene following host passage than seen when the transposase activity was encoded in capped RNA. This suggests that in *S. ratti*, co-transfected helper plasmids designed for in situ expression of the *piggyBac* transposase constitute a more efficient means of providing this activity than in vitro translated capped RNA. By contrast, co-transfection with capped RNA encoding the transposase gives efficient *piggyBac*-mediated integration of transgenes in *Schistosoma mansoni*, whereas expression of the transposase from co-transfected helper plasmids does not [39]. Co-transformation of *S. ratti* with donor plasmid pPV254, which lacks the gypsy insulator sequences, and helper plasmid pPV402 (Table 1, Experiment 5) yielded transgene-expressing individuals in the F2 generation. This indicates that although the gypsy insulator sequences effectively resist positional silencing of transgenes in *Drosophila* [34], they are not required for sustained transgene expression in *S. ratti*. This result further supports the hypothesis that chromosomal integration of transgenes is the essential factor provided by this system.

**Table 1:** Heritable transgene expression and establishment of stable transgene-expressing lines in *S. ratti* as a function of various pairings of donor and helper plasmids incorporating elements of the *piggyBac* transposon.

Experiment	Donor Construct	Helper	No. P0 Injected	No. F1 Larvae GFP+ (No. Screened)	% F1 Expression	No. F2 Larvae GFP+ (No. Screened)	% F2 Expression	Stable line obtained
1	pPV356	None	160	72 (1854)	3.4	0 (1194)	0	No
2	pPV356	Transposase mRNA	195	235 (3716)	6.3	4 (14278)	0.03	No
3	pPV356	pPV402	205	70 (784)	8.9	34 (8511)	0.4	Yes
4	pPV356	pPV402	50	15 (428)	3.5	19 (867)	2.2	Yes
5	pPV254	pPV402	110	45 (1105)	4.1	56 (2282)	2.5	Yes

### Transgene-expressing *S. ratti* of the F2 generation can found stable lines with sustained inheritance and expression of transgenes

**Figure 2:**
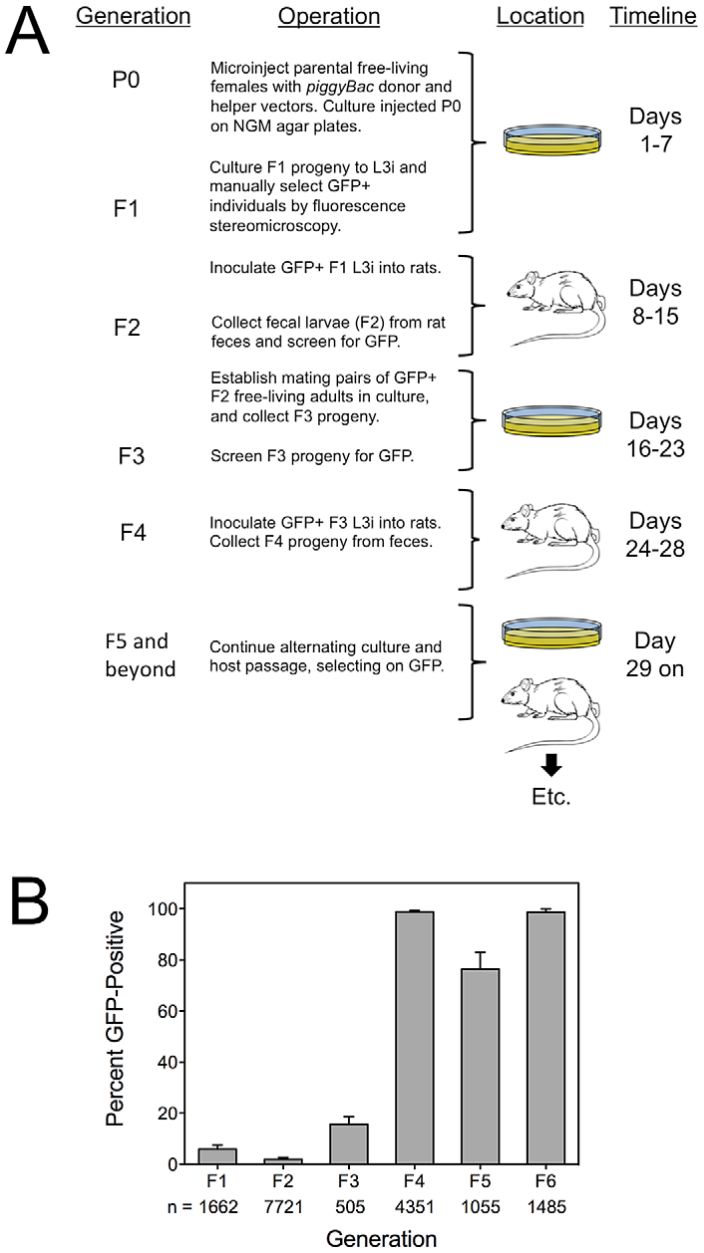
Stable transgenic lines of *S. ratti* are established by microinjection of nucleic acid constructs followed alternating rounds of host and culture passage, selecting on expression of a fluorescent reporter gene product. **A**) Diagram of major steps in isolation of stable transgenic lines of *S. ratti*. Initial gene transfer into parental (P0) free-living females is by gonadal microinjection of donor vectors alone or in combination with helper vector or capped transposase encoding mRNA. F1 larvae are derived in culture and screened for GFP expression. Transgene expressing individuals are reared to infective L3 (L3i) and inoculated into rats to establish as parasitic females. F2 progeny released in the feces of these rats are screened for transgene expression and positive larvae reared in culture to free-living males and females and allowed to mate. F3 progeny arising from these crosses are reared to L3i in culture in inoculated into rats. F4 progeny arising in the feces are screened for reporter transgene expression and used for further alternating rounds of culture and host passage with continued selection on GFP. The timeline indicates the intervals in days following microinjection of parental worms in which each generation is isolated up to the F5 when transmission and expression are generally stabilized. **B**) Frequency of transgene expressing progeny by generation during selection of stable transgenic lines of *S. ratti*. Data are mean (± one standard deviation) percentages of reporter transgene-expressing individuals in each generation of alternating host and culture passage with selection on GFP (see panel A). Means are derived from three independently established lines (PV2, PV3 and PV4). Sample sizes (**n**) indicated below the abscissa are totals of worms examined from all three lines. Statistics: in panel B, 2-way ANOVA, performed using Prism ver. 5.0c (GraphPad Software, Inc., La Jolla, California, USA), revealed a highly significant effect due to generation (P<0.0001) but no significant effect (P = 0.2) due to donor plasmid.

Having observed the first transgene expression in the F2 generation of *S. ratti*, we asked whether these F2 individuals could found stable transgenic lines. We did so using the protocol illustrated in Figure 2A. Briefly, parental free-living females of *S. ratti* were transformed by gonadal microinjection with *Ss-act-2::gfp* in *piggyBac* vectors as described previously for plasmid vectors [24], [25]. F1 progeny of microinjected females were reared in culture and screened for reporter expression by fluorescence stereomicroscopy. GFP+ progeny were cultured to infective L3 (L3i) and inoculated into rats. F2 progeny arising in feces of these rats were screened for GFP expression and hand selected. These F2 individuals were cultured to mating pairs of free-living adults, and their progeny (F3) were reared to L3i, selected for expression of the transgenes, and inoculated into rats. *S. ratti* eggs and first-stage larvae in the feces of these rats constituted the F4 generation. This pattern of alternating culture, selection for expression and host passage was then repeated for subsequent generations of selection.

The results (Fig. 2B), expressed as the percentage of GFP+ individuals in the population screened within each generation, demonstrate derivation of stable lines with virtually 100% inheritance and expression of transgenes within 5 generations of selection. Numbers of individuals available for passage in the F1 and F2 generations were low for all three lines, but could be amplified owing to the high susceptibility of laboratory rats to *S. ratti* infection. The decline in the proportion of GFP-positive larvae in the F5 generation is noteworthy. In our selection scheme (Fig. 2A), the F5 generation arises from crossing of GFP-positive free-living males and females in culture. It is possible that some heterozygous individuals remain in the F5 generation and that the observed decline in the frequency of GFP-expressing individuals in the F5 resulted from segregation of homozygous wild-type individuals among the progeny of crosses between heterozygotes or between heterozygotes and homozygous transgenic worms. We are continuing to maintain these lines by serial passage. Line PV2 is currently in the F10 and exhibits GFP fluorescence in 100% of individuals; PV3 is in the F10 with expression in 98.6% of individuals, and PV4 is in the F8 with expression in 99.4% of individuals. While expression rates in PV3 and PV4 may reflect remaining heterozygosity in these lines, they could also be due to diminished levels of expression or viability in small proportions of the worms examined by fluorescence stereomicroscopy. We have cryopreserved transgenic L3i from each line as described [40] (at −80°C instead of in liquid nitrogen) and have thawed viable late-passage worms with a recovery rate of approximately 10% (data not shown). All thawed L3i were transgenic, as evidenced by expression of GFP, indicating that in the lines observed, integration of the transgenes did not affect parasite fitness in a way that would render them more susceptible to damage during cryopreservation.

### Expression patterns of a reporter transgene in stable lines are consistent with those seen in transiently transformed *Strongyloides* spp

**Figure 3:**
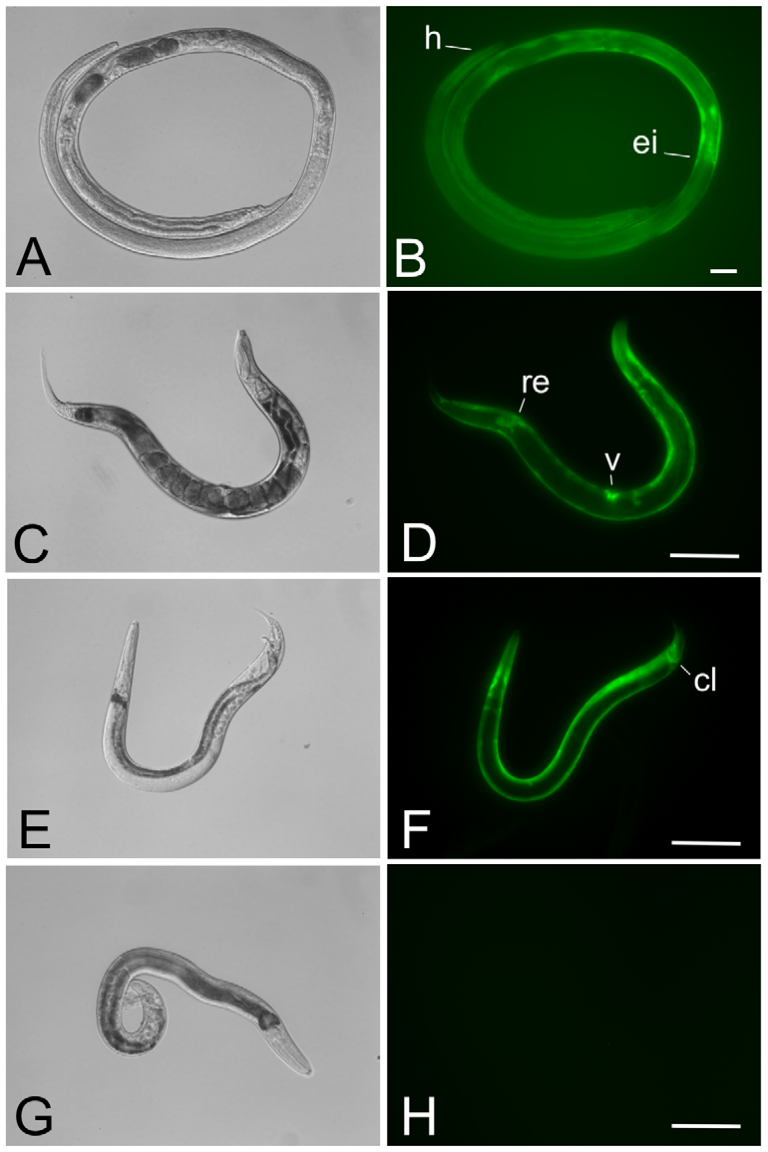
Parasitic and free-living adults from a stable transgenic line of *Strongyloides ratti* express GFP in the body wall-specific manner expected for the *Ss-act-2*prom::*gfp* transcriptional reporter. Typical patterns of GFP expression in parasitic female, free-living male and free-living female *S. ratti* expressing the integrated reporter transgene encoded in pPV356. (**A, B**) DIC and fluorescence images, respectively, of a parasitic female *S. ratti* from integrated line PV2. Note expression predominating in the body wall up to the level of the esophageal/intestinal boundary (**ei**). Position of the head is indicated (**h**). (**C, D**) DIC and fluorescence images, respectively, of a free-living female *S. ratti* from integrated line PV2. Note uniform expression throughout the body wall with additional loci of expression in the vulva (**v**) and rectum (**re**). (**E, F**) DIC and fluorescence images, respectively, of a free-living male *S. ratti* from transgenic line PV2. Note uniform expression in the body wall with additional expression in the cloaca (**cl**). (**G, H**) DIC and fluorescence images, respectively, of a non-transformed free-living male *S. ratti*. The fluorescence image in panel H was exposed for a period≥exposure times in panels B, D and F. Scale bar = 200 µm in all panels.

The expression pattern of the transcriptional reporter *Ss-act-2*prom::*gfp*::*Ss-era-1* 3′ in parasitic females from a stable integrated line (PV2) of *S. ratti* were similar to that reported previously [24] for F1 larvae of *S. stercoralis* transformed with a conventional plasmid vector (pAJ08) and presumably expressing this transcriptional reporter from a multi-copy episomal array. In general, parasitic females from line PV2 exhibited a zone of fluorescence in the body wall extending posteriorly from the junction between pharynx (esophagus) and intestine to a level approximately even with the posterior margin of the uterus (Fig. 3A, B). Retention of the body-wall specific pattern of *Ss-act-2*-regulated GFP expression in stable *S. ratti* transformants is consistent with previous findings that promoters derived from *S. stercoralis* drive reporter transgene expression in identical or highly similar anatomical patterns in *S. ratti* [26]. Moreover, this observation suggests that expression patterns of transgenes integrated into limited numbers of chromosomal sites in *Strongyloides* spp. will be consistent with patterns resulting from over expression of the same transgenes from high copy number episomal arrays.

The expected body wall specific pattern of the *Ss-act-2*prom::*gfp* reporter was also seen in free-living females (Fig. 3C, D) and free-living males (Fig. 3E, F). In addition to uniform expression throughout the body wall, additional loci of GFP expression were seen in the vulva and rectum of free-living females (Fig. 3D) and in the cloaca of free-living males (Fig. 3F). No fluorescent signal could be detected from non-transformed free-living male *S. ratti* (Fig. 3G, H). Owing to the failure of non-integrated transgenes to express beyond the F1 generation, free-living adult *Strongyloides* expressing transgenes had not been observed prior to the establishment of stable integrated lines in *S. ratti*.

### Transgene sequences in stable lines generated by the *piggyBac* transposon system are integrated in TTAA sites in the genome of *S. ratti*

**Figure 4:**
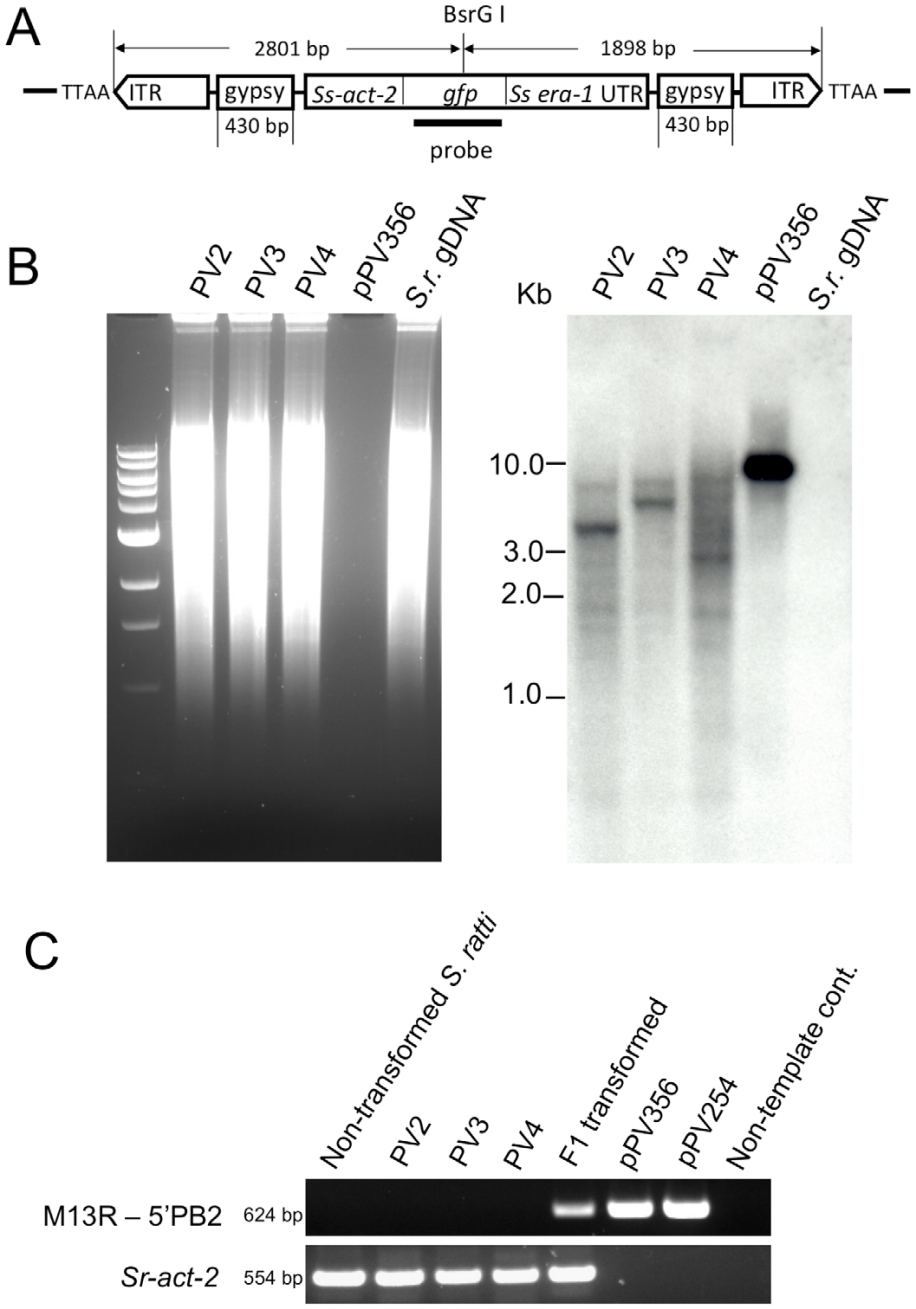
Transgene specific sequences are widely distributed in restriction digests of genomic DNA from three stable lines of transgenic *S. ratti*. Southern hybridization of genomic DNA (gDNA) of *S. ratti* from stably transformed lines probed for the *gfp* coding sequence. **A**) Transgene diagram showing position of the probe used for Southern hybridization analysis and the single restriction site for BsrGI, the enzyme used for restriction digestion of gDNA. **B**) Southern blot of BsrGI digests of gDNA from pooled free-living adults from three independent integrated lines (PV2, PV3 and PV4). The positive control lane is a blot of a BsrGI digest of donor plasmid pPV356, and the negative control is a blot of a BsrGI digest of gDNA from non-transformed *S. ratti* free-living adults (*S.r.* gDNA). Note *gfp* hybridization signals in multiple restriction fragments in gDNA from each of the three integrated lines. A single hybridizing band of 8.1 kb appears as predicted in the positive control digest of pPV356. No *gfp*-specific signal is detected in the negative control digest of gDNA from non-transformed *S. ratti*. **C**) Gel analysis of PCR products from genomic DNA templates from non-transformed control parasites, parasites from each of three stable lines, PV2, PV3 and PV4, with integrated, stably expressed transgenes and F1 progeny of free-living female worms microinjected with donor plasmid pPV356 and helper plasmid pPV402. Also included are PCR products from control reactions with plasmids pPV356 and pPV254 as templates and from a reaction from which template was omitted. Upper gel image depicts 624 bp amplification products resulting from a forward primer hybridizing to the M13 reverse priming site in the vector and a reverse primer hybridizing within the transposon sequence. Lower gel image is a loading control showing the expected 554 bp amplification product from reactions with primers specific for the constitutively expressed cellular actin-encoding gene *Sr-act-2*.

We inferred chromosomal integration of the reporter transgene in *S. ratti* from a Southern blot of a BsrGI restriction digest of genomic DNA from each transgenic line which was hybridized with a *gfp*-specific probe (Fig. 4A). We detected *gfp*-specific sequences in multiple fragments with various molecular weights in the genomic DNA of each line (Fig. 4B). Based on the presence of only one BsrGI site in the vector, occurring within the *gfp* coding sequence, the smallest predicted transgene-containing restriction fragments of the genome would be 1898 bp for worms transformed with the donor vector pPV356, which contains the 430 bp gypsy insulator sequences or 1468 bp for worms transformed with donor vector pPV254, which does not contain these sequences (Fig. 4A). The Southern hybridization patterns seen for PV2, PV3 and PV4 (Fig. 4B) are generally consistent with this prediction, showing a multiplicity of bands hybridizing with the *gfp*-specific probe in the range of approximately 1.8 kb to 7.5 kb. By contrast, this diverse banding pattern is not consistent with the uniform 8084 and 6758 bp fragments that would be expected to result from BsrGI digestion of episomal arrays made up of the donor vectors pPV356 and pPV254, respectively.

Chromosomal integration was confirmed by splinkerette-PCR, which allowed sequencing of the genomic regions bounding unique transgene insertions in five contigs of the *S. ratti* genome (Table 2). The current draft genome assembly for *S. ratti* (http://www.sanger.ac.uk/resources/downloads/helminths/strongyloides-ratti.html) allows assignment of three of these contigs to Chromosome I. Further analysis, in which we subjected 2 kb sequences flanking each of the five identified transposon insertion sites (Table 2) to BlastN/BlastX searching against the non-redundant nucleotide/protein sequences, revealed three insertions in coding regions. All insertions were at TTAA sites (Table 2) as has been found in other studies of the *piggyBac* transposon [21], [39], [40]. Insertion PV2-2 (Table 2) is near the 5′ end of a sequence predicted to encode a peptide that is 51% identical to the hydroxyacylglutathione hydrolase in *Loa loa* (Accession XP_003137817). Insertion PV3-1 is within the coding sequence of a 28S ribosomal RNA gene that has 98% nucleic acid identity with AB205054 in *S. stercoralis*, and insertion PV4-1 is within a gene sequence predicted to encode a peptide with 68% identity to hypothetical protein T13G4.3 in *Caenorhabditis elegans* (ADD13552.1) (XP_001894192.1). Insertions PV2-1 and PV2-3 were in intergenic regions. Although the wide molecular weight ranges of restriction fragments of gDNA with *gfp*-specific sequences in all three lines (Fig. 4B) are suggestive, the breadth of transgene integrations throughout the genome of *S. ratti* cannot be assessed accurately from the few sequenced integration junctions reported here. Additionally, splinkerette-PCR, the technique used to sequence and map integration boundaries, is biased to the restriction sites of the enzymes used to digest the genomic DNA. In future studies, high-capacity genome sequencing could be brought to bear, as done previously with *Salmonella typhi* [41], to determine whether the pattern of *piggyBac* insertions in *S. ratti* is consistent with the observed broad patterns of *piggyBac* insertions in the genomes of *Drosophila melanogaster* [42] and the parasitic trematode *S. mansoni* [39]. The fact that three of the five insertions we sequenced were in coding sequences in the in *S. ratti* genome is consistent with the *piggyBac* transposon's documented propensity to integrate into the coding regions of genes in other organisms [42], [43]. This observation underscores the potential of the *piggyBac* transposon system as a tool for insertional mutagenesis in unbiased forward genetic investigations with *S. ratti*.

**Table 2:** Genomic integration sites for reporter transgenes and their flanking sequences in three stable transgenic lines of *Strongyloides ratti*.

Stable line	Donor vector	Insertion	Integration junction accession	Contig	Chr.^a^	Location	5′ sequence junction	Target site	3′ sequence junction
PV2	pPV356	PV2-1	JX070935	74278	I	172601	ACTTTAACGTTGTCATGATTTTTTT	TTAA	ATTTCATTATACTATTTTAAAAGTA
“	pPV356	PV2-2	JX070931	74996	I	155901	GGTGGTGGAGATAAAATTAGTTATG	TTAA	TAAAATTGTTAAAGATGGTGATGAA
“	pPV356	PV2-3	JX070932	75336	ND^b^	3211	TTCGATATCTCTTGTTACTTTTCAA	TTAA	AAAAAAAAATATTTTTTAAGGCAAA
PV3	pPV356	PV3-1	JX070933	75034	ND^b^	3303	GTATCCAAAGTGCTTGTTATATTAC	TTAA	AAAGTTAATGGAAGCATAATAAATT
PV4	pPV254	PV4-1	JX070934	75488	I	603672	ATAAGAACACGTCCTAAGTGAAATC	TTAA	TGCTGTTCGAGGATGAAAATTTTCA

Contig identities, insertion locations within contigs and, where possible, chromosome numbers are derived from the draft *S.ratti* genome sequence.

a Chromosome.

b Not determined.

Estimates of relative copy number of integrated transgenes derived by qRT-PCR were similar in the three independent lines with mean relative copy number ranging from 37 to 51 per genome (Table 3). These means were not significantly different (P>0.05), indicating that the transformation protocol results in a consistent number of transgene integrations. To ensure that the estimates of relative copy number of integrated transgenes did not reflect sequences in episomal arrays, we conducted PCR on gDNA from each stable line using forward and reverse primers hybridizing within the M13 reverse priming site of the vector and within the transposon sequence respectively. These primers did not yield a product from gDNA of the stable integrated lines (F8 generation), but did from the F1 progeny of free-living female worms microinjected with donor (pPV356) and helper plasmids (Fig. 4C). This suggests that donor plasmids are transmitted to the F1 generation but are lost during passage. We conclude, therefore, that our estimates of relative transgene copy number reflect numbers of chromosomal integrations. While random integration of large numbers of transposons would be desirable in unbiased studies aimed at gene tagging or disruption, integration of large numbers of transgene copies in focused studies of gene function is less than desirable given the risk of non-physiologic effects of over expression and non-specific effects resulting from insertional mutagenesis. It is noteworthy in this regard that no unusual or unexpected phenotypes have been evident among worms from the three stable lines in the present study. A technique for single-copy integration of transgenes in *C. elegans* based on the Mos-1 transposon system has been developed recently [44] and could provide a strategy for achieving similar results in *Strongyloides* spp. in the future.

**Table 3:** Analysis of relative transgene copy number via qRT-PCR for stable transgenic lines of *S. ratti* transformed with the *piggyBac* transposon system.

Stable line	Donor construct	Assay replicate	Ct e (Mean ± SD)^a^	Ct t (Mean ± SD)^a^	ΔCt	ΔΔCt	2^−ΔΔCt^	Relative copy number (Mean ± SD)^b^^,^ c
PV2	pPV356	1	19.95±0.26	21.45±0.04	1.49	−5.62	49	
		2	19.53±0.05	21.36±0.09	1.83	−5.72	53	51±2
		3	19.86±0.16	21.10±0.24	1.24	−5.66	51	
PV3	pPV356	1	20.49±0.12	22.31±0.10	1.82	−5.29	39	
		2	20.08±0.15	22.23±0.07	2.20	−5.35	41	37±6
		3	20.19±0.25	22.18±0.10	1.99	−4.92	30	
PV4	pPV254	1	21.02±0.19	22.44±0.11	1.42	−5.69	52	
		2	20.50±0.25	22.28±0.07	1.78	−5.77	55	48±9
		3	20.52±0.13	22.21±0.06	1.69	−5.21	37	
Control^d^	none	1	23.97±0.18	31.08±0.15	7.11	0	1	
		2	23.91±0.11	31.46±0.30	7.55	0	1	1
		3	24.88±0.20	31.79±0.20	6.91	0	1	

a Calculated from three technical replicates within the assay.

b Calculated from three replicate assays.

c Differences between mean relative copy number for stable lines not significant by one-way ANOVA (P>0.05).

d Wildtype *S. ratti*.

Note that a stable, GFP-expressing line was established using the donor plasmid pPV254, which lacks the gypsy insulator sequences, (Fig. 1; Tables 1 and 2). Furthermore, the proportion of GFP expressing individuals among all larvae screened did not vary significantly as a function of donor construct (Fig. 2B). Together, these observations indicate that under conditions pertaining in the present study, where integrations appeared to occur widely throughout the genome, with mean relative copy numbers ranging from 37–51 per genome, the gypsy insulator sequences are not required for stable expression of transgenes in integrated lines of *S. ratti*. However, the gypsy sequences were originally characterized as resisting positional silencing effects in *Drosophila*, which vary significantly based on the site of transgene integration [34]. Although positional silencing of transgenes has not been documented in *S. ratti*, it is possible that, as we attempt to achieve integrations at lower copy number or to adapt methods for single copy transposon-mediated transgenesis [44] to this parasite, such positional effects will manifest themselves and be a significant factor in stable transgene expression. In such cases it may be prudent to include the gypsy sequences as data from the present study also indicate that they do not adversely affect the efficiency of integration.

In summary, we have developed a transposon-based system for integration of transgenes into the chromosomes of *Strongyloides* spp. and for the establishment of stable transgenic lines. *Strongyloides ratti* lends itself particularly well to establishment of stable integrated lines owing to its efficiency of infection in laboratory rats. Three such integrated lines have been established in *S. ratti*, and boundary sequences of multiple unique transgene integration sites have been mapped. The possibility of introducing heritable, stably expressed transgenes into a parasitic nematode is an advance that offers important new opportunities to study gene function in these organisms. Perpetuation of stable transgenic lines will greatly facilitate functional approaches involving expression transgenes with dominant interfering or activating mutations as was recently done with very limited samples of transiently transformed *S. stercoralis* [45].

Transposon mediated transgenesis will also be a useful adjunct to other methods of genetic analysis such as RNAi. The majority of animal parasitic nematodes exhibit very limited sensitivity to exogenously administered RNAi [46]. RNAi targets in *Haemonchus contortus* that are located on cell surfaces in contact with the worm's external environment show greater RNAi sensitivity than internal targets [47], suggesting that this, and perhaps other parasitic species, are deficient in mechanisms that spread administered dsRNA from cell to cell. Endogenously expressing interfering RNAs from transgenes in stably transformed worms could be a means of surmounting barriers to uptake and transport of administered dsRNA. This approach has shown promise in transiently transformed schistosomes [48], [49], [50]. Furthermore, by stable heterologous expression of dsRNA transporters such as *C. elegans* SID-2 [51] or of Argonaut proteins such as RDE-4, it may be possible to complement specific deficiencies in RNAi processing suggested by recent transcriptomic studies in parasitic nematodes [33], [52].

With regard to the use of a transposon-based approach to transgene integration *per se*, improvements in the efficiency of transposition will enable such techniques as transposition induced gene knockouts [42], exon and enhancer traps [53], and excision-based site directed mutagenesis [54] to further the genetic analysis of these species. The use of transposon integration sites as genetic markers [55] could aid in the assembly of chromosome scaffolds for the ongoing genome sequencing projects for these worms.

While the present finding constitutes a substantial advance in transgenesis for *Strongyloides* and related genera, its impact would be greater if the technology could be adapted to other parasitic nematodes of medical or agricultural importance. We envision two potential hurdles, the lack of parthenogenetic parasitic females and challenges in gene transfer, that would need to be surmounted in order to accomplish this. The property of parthenogenesis in parasitic females of *Strongyloides* spp. [28] represents an advantage in deriving transgenic lines by host passage in that it makes it possible to obtain large numbers of progeny from parasitic females without the presence of males. Moreover, progeny from individual parthenogenetic females are clonal [56] and so matings in subsequent free-living generations are effective selfings of the parasitic female parent, further hastening the selection of homozytotic transgenic parasites. These advantages would not be available in other parasitic nematode taxa of interest, which are dioecious. Nevertheless, if sufficient numbers of transgenic progeny from the F1 and subsequent generations, comprising both male and female larvae, and a sufficiently well adapted host-parasite system in which to carry out line selection were available, these conditions could combine to enable the establishment of stable transgenic lines of dioecious parasitic nematodes, albeit with a more lengthy and labor intensive process than in *Strongyloides* spp. The ability to transfer genes into *Strongyloides* spp. and related genera by gonadal microinjection of free-living females [27], [57] also constitutes a major advantage in undertaking transgenesis that will be unavailable in the majority of parasitic nematode species that cannot undertake full generations of free-living development. However, larval stages of animal parasitic nematodes that arise in the extrinsic environment or in arthropod vectors can be cultured transiently and may be susceptible to gene transfer by other routes. This potential was recently demonstrated emphatically in the heritable transformation of *Brugia malayi* by chemically mediated gene transfer [58]. Gene transfer in this study was achieved by incubating developing third-stage larvae with co-precipitates of the DNA vector with calcium phosphate. This method succeeded with larvae that were inoculated along with the co-precipitate into the peritoneal cavities of susceptible gerbils but not with larvae cultured in vitro with co-precipitate, even in a culture system that promoted L3–L4 molting. This finding may indicate that completion of a molt cycle with subsequent remodeling of the nascent cuticle, which occurs in the host, but may not occur normally in cultured *B. malayi* L3, is necessary for sufficient uptake of the DNA-CaP0_4_ co-precipitate. In any case, this method of chemically mediated gene transfer into an accessible larval stage represents one that, in theory, could be adaptable to transposon mediated integration of transgenes into a broad range of parasitic nematodes of medical and veterinary importance.

## Materials and Methods

### Ethics statement

Rats and gerbils were used for parasite strain maintenance and host passage of transgenic *S. ratti* in this study. These procedures were approved by the Institutional Animal Care and Use Committee (IACUC) of the University of Pennsylvania (Protocol No. 803511). This protocol, as well as routine husbandry care of the animals, was in strict accordance with the *Guide for the Care and Use of Laboratory Animals of the National Institutes of Health*.

### Worm strains, animal infection and maintenance of transgenic lines

The ED321 strain of *S. ratti* was maintained in rats and gerbils and cultured as described [59]. Free-living adult *S. ratti* were isolated via the Baermann funnel technique from charcoal coprocultures maintained at 22°C for 48 hours. The worms were washed twice with sterile deionized water to reduce carryover of fecal bacteria and plated on Nematode Growth Medium (NGM agar) plates with lawns of *Escherichia coli* HB101. Agar plate cultures of *S. ratti* were incubated at 22°C unless otherwise noted. Rats were infected with *S. ratti* by subcutaneous injection of infective larvae (L3i). Naïve rats are highly susceptible with *S. ratti*, frequently developing patent infection after inoculation of only one or two infective larvae [60], [61]. This makes them the host of choice for early generations of passage during isolation of stable lines, when very few transgenic L3i are available. Rats, however, mount a vigorous protective immune response to *S. ratti* infection, and eliminate their infections about one month following inoculation [62]. Gerbils are also susceptible to infection with *S. ratti* but require 50–100 L3i to establish a patent infection. However, gerbils do not expel their infections as rapidly as rats and so allow maintenance for 6 months or more in individual animals [63]. This makes them the hosts of choice for maintaining established stable transgenic lines, where numbers of L3i are not limiting. Therefore, once established by 3 or 4 generations of alternating passage through free-living culture, selection and rat infection, stable transgenic lines of *S. ratti* were maintained by serial passage in gerbils thereafter. Cohorts of L3i from each stable line were also cryopreserved as described [40] and the viability of thawed parasites verified by observation of patency following gerbil inoculation.

### Plasmid constructs

We created donor vector pPV254 (Fig. 1A; GenBank accession number, JX013636) by excising the previously described reporter cassette, *Ss-act-2p*::*gfp*::*Ss-era-1* 3′ UTR, from pAJ08 [24] (Addgene Plasmid #14912) with HindIII and EagI (Unless otherwise noted, all restriction and other DNA processing enzymes used in this study were obtained from New England Biolabs) and ligating with T4 DNA Ligase into the *piggyBac* donor vector pXL-BacII [38] containing inverted terminal repeats (ITRs) of the *piggyBac* transposon and essential internal sequences surrounding a multi-enzyme cloning site (MCS), which was cut with the same restriction enzymes.

As a precaution against epigenetic silencing, the gypsy retroviral insulator sequences, which retard positional silencing effects in *Drosophila* [34], were introduced at the 5′ and 3′ ends of the expression cassette in donor vector pPV254 to create the new donor vector pPV356 (Fig. 1B; GenBank accession number, JX013635). Tandem 430 bp gypsy insulators were excised from plasmid pCa4B2G, a kind gift from Norbert Perrimon, Harvard University School of Medicine, by digestion with Sac I and Not I. The fragment containing the gypsy insulators was isolated by gel purification and blunt ended by Pfu turbo DNA Polymerase (Stratagene). Plasmid pPV254 was then digested with Hind III and Xba I, and the larger (3970 bp) fragment, which contained the *piggyBac* ITRs but lacked the expression cassette, was isolated and blunt ended as described above. These two fragments were then ligated to yield a *piggyBac* plasmid with two gypsy insulators. Subsequently, a multi-cloning site (MCS) (GGGCCCACTAGTCGGCCGGAATTCCTAGGACCGGTACCCTGCAGAAGCT) was cloned into the plasmid between the gypsy insulators to create a multi-purpose insulated *piggyBac* vector. Finally, the expression cassette, *Ss-act-2*p::*gfp*::*Ss-era-1* 3′UTR from pAJ08 was cloned into the MCS by digestion with Xba I and Hind III and ligation with T4 DNA Ligase to yield pPV356.

pPV402 (Fig. 1C; GenBank accession number, JX013634), the *piggyBac* transposase helper vector, was made by excising the *piggyBac* transposase coding sequence from pPV257 (see below) with the restriction enzymes AgeI and AvrII and cloning them into the pAJ50 vector [24] (Addgene Plasmid #14918) from which the *gfp* coding region had been removed with the same restriction enzymes. This yielded pPV402, in which the *piggyBac* transposase is expressed under the control of the *Ss-rps-21* promoter, which drives expression of transgenes in all tissues including the germline of microinjected P0 female worms and in F1 transformants [24].

To prepare in vitro transcribed synthetic mRNA for microinjection in lieu of a helper plasmid, pPV257 (Fig. 1D; GenBank accession number JX017375) was made by first amplifying the *piggyBac* transposase coding sequence from pBSII-IE1-orf (http://piggybac.bio.nd.edu/) with the PCR primers HelpKpnIAgeIF (CCTGCAGCCCGGGTACCGGTATATAATAAAATGGG) and HelpAvrIIEcoRIR (GTGGCGGCCGCTGAATTCCTAGGGGATCCAAATTC) using the Pfu Turbo DNA polymerase (Stratagene). The PCR amplicon was cloned into the pCR-Blunt II-TOPO vector (Invitrogen) to yield pPV257. This construct was linearized with AvrII and used for in vitro transcription from the T7 promoter of the parental pCR-Blunt II-TOPO plasmid using the mMessage mMachine T7 Ultra kit (Ambion, Austin, TX, USA), which includes capping and poly-A tailing reactions, according to the manufacture's instructions.

### Microinjection of *Strongyloides ratti*

Constructs were microinjected into one arm of the syncytial gonad of free-living *S. ratti* females as described for *C. elegans* [31]. Microinjected worms were recovered on NGM ager plates with lawns of *Escherichia coli* HB101 and cultured at 22°C with free-living males. At 48 h following injection, parental (P0) females and their broods were observed, and both total and GFP-positive F1 eggs and larvae were counted. Over the ensuing week, GFP positive third stage larvae (L3i) of the F1 generation were reared, manually selected and stored in BU buffer [64] for animal inoculation. Basic steps of this procedure and their timing are illustrated in Fig. 2A. Frequencies of GFP expression in worms screened from each of three transgenic lines, including one transformed with donor vector pPV254 and two transformed with pPV356, were compared statistically as detailed in the caption to Fig. 2. The criterion for significance was P≤0.05.

### Genomic Southern blotting

Genomic DNA was extracted from control and transformed *S. ratti* from the F8 generation of each stable line using the Gentra Puregene Tissue Kit (QIAGEN). Genomic DNA and donor plasmid pPV356 were digested with BsrGI. Each donor vector, pPV254 and pPV356, contains only one BsrGI site. BsrGI was selected because the location of its single cut site within the *gfp* coding sequence would result in two fragment ends that hybridize with the probe for each integration event, thus increasing the intensity of the signal in our Southern blot.

Restriction fragments were separated by electrophoresis through 1% agarose, transferred to nylon membranes and fixed there by cross linking with UV light using a UV Stratalinker 1800 (STRATAGENE). A probe homologous to a 723 bp segment of the *gfp* coding sequence was generated by PCR using primers: GFP-metF (5′ ATGAGTAAAGGAGAACTTTTC 3′) and GFP-702R (5′ ATCGCCAATTGGAGTATTTTGT 3′). The PCR product was purified by agarose gel electrophoresis followed by gel extraction with the QIAquik kit (Qiagen). The probe was labeled with digoxigenin–dUTP using DIG High Prime DNA labeling and detection starter kit II (Roche). The DIG-labeled *gfp* gene probe was hybridized to Southern blots at 42°C overnight with gentle agitation. Membranes were washed at high stringency, the probe detected immunologically with CSPD and the resulting blot imaged on X-ray film (Kodak).

### Splinkerette-PCR

Chromosomal integrations of transgenes were further confirmed and mapped by splinkerette-PCR (spPCR) using a modification of Splinkerette Protocol S1 [65]. Genomic DNA was extracted from free-living adult worms of the transgenic lines using the Gentra Puregene Tissue Kit (QIAGEN). Purified DNA (∼100 ng) was digested with BstYI, BamHI or Bgl II for 2 h in a total volume of 15 µl for each reaction. Digested DNA was ligated with T4 Ligase to annealed splinkerette oligonucleotides (SPLNK-GATC-TOP and SPLNK-BOT in protocol S1) with GATC sticky ends for 8 h at 16°C in a total volume of 30 µl. Following ligation of gDNA restriction fragments to splinkerette oligos, those containing transgene integrations were identified, and integration boundaries recovered by primary and nested spPCR using appropriate combinations of primers Splnk-1 and Splnk-2, which target the ligated splinkerette oligos, and 3′PB1 and 5′PB1, which target the 3′ and 5′ ends of the transgene coding sequence, respectively. For primary spPCR, Splnk-1 was paired with either 3′PB1 or 5′PB1, and amplification was carried out with Phusion Polymerase and approximately 20 ng of splinkerette-ligated genomic DNA as template. Thermal cycling conditions were 98°C for 1 min followed by 30 cycles of 98°C for 20 sec, 55°C for 15 sec and 72°C for 2 min, with a final extension at 72°C for 10 min. Nested PCR was carried out with Splnk-2 paired with 3′PB2 or 5′PB2 and a 1∶20 dilution of spPCR products as template. Amplification with Phusion Polymerase was carried out using the same thermal cycling conditions detailed for primary spPCR. Primary and nested PCR products were analyzed by 1% agarose gel electrophoresis. After treatment using Shrimp Alkaline Phosphatase (United States Biological) and Exonuclease I, nested PCR products were sequenced using primers, 3′PB SEQ and 5′PB SEQ, targeting the 3′ and 5′ ends of the transgene coding sequence, respectively.

### Estimation of relative copy numbers of integrated transgenes

Relative numbers of transgene copies integrated into each of the stable lines were estimated by real-time PCR using gDNA as template as described [66]. Relative copy number was expressed as the mean of Ct (2^−ΔΔCt^) determined from three independent amplifications. Transgene DNA was quantified using primers specific for the coding sequence of *gfp*: gfp-F (5′ACCCTTGTTAATAGAATCGAG3′) and gfp-R (5′TCAATGTTGTGTCTAATTTTGAAG3′). The gene *aap-1*, which encodes the ortholog of the phosphoinositide 3-kinase (PI3K) *p50*/*p55* adaptor/regulatory subunit, exists as a single copy in *S. ratti* genome and so was selected as a reference gene for relative copy number determination. Primers aap-1F (5′TACCAGAAGATGATGTAGATGC3′) and aap1R (5′AGTTTATTGACTTTAGTTGTCAATG3′) were designed based on the sequence of *S ratti aap-1*. The size of amplicons from the *aap-1*- and *gfp*-specific primers were identical at 210 bp. Real-time PCR amplification was performed with a 7500 Fast Real-time PCR system using the SYBR Green Master Mix (Applied Biosystems, Foster City, CA, USA). 10 ng of genomic DNA template were added in a total volume of 20 µl for each reaction. Each sample was set up in triplicate to give three technical replicates of the reaction. Reaction conditions were: start at 50°C for 2 min, initial denaturation at 95°C for 10 min, followed by 40 cycles of 95°C for 15 sec, 56°C for 1 min. The dissociation curve was run at 56°C for 60 min. Three independent amplifications (denoted “measuments” in Table 3) were performed on gDNA from each line with three technical replicates per amplification. As stipulated previously [66], quality control criteria for reliable results were a Ct<25 for an amplicon and a standard deviation <0.3 for technical replicates in an amplification.

Relative copy number determinations for the integrated transgenes could be subject to inteference from transgene sequences in extrachromosomal arrays formed by the donor vector. To assess this eventuality, we conducted diagnostic PCR reactions with primers designed to detect vector sequences, presumed to be in extrachromosomal arrays, persisting in worms from the three stable transgenic lines. We preformed PCR using a pair of donor vector-specific primers for diagnostic PCR: M13 reverse, hybridizing with the M13 reverse priming site in the vector, and 5′PB2 (see splikerette PCR protocol above) hybridizing within the *piggyBac* transposon sequences of both vectors. These primers amplify an identical 624 bp product from both donor vectors used in the study. The primers SrAct-2F (5′-TGGAGATGAGGCCCAATCC-3′) and SrAct-2R(5′-GTGATGAAGATGAAGCAGCTGTG-3′) were designed to amplify a 554 bp fragment of endogenous gene *Sr-act-*2, which was used as loading control. The DNA templates in the amount of 5 ng were used in the PCR reaction, which was carried out with Phusion Polymerase. Thermal cycling conditions were 98°C for 1 min followed by 25 cycles of 98°C for 20 sec, 57°C for 15 sec and 72°C for 40 sec, with a extension at 72°C for 10 min.

### DIC and Fluorescent microscopy

Screening for transgenic larvae based on GFP fluorescence was carried out with a Olympus SZX12 stereomicroscope equipped with coaxial epifluorescence. Fine-scale examination of transgene expressing worms was carried out with an Olympus BX60 compound microscope with Nomarski Differential Interference Contrast (DIC) optics and epifluorescence (Olympus America Inc., Center Valley, Pennsylvania, USA). Images from the Olympus BX60 were captured with a Spot RT Color digital camera and processed using either the Spot Advanced image analysis software package (Diagnostic Instruments, Inc., Sterling Heights, Michigan, USA) or Adobe Photoshop 7.0. Image-processing algorithms such as contrast and brightness adjustments were always applied in linear fashion across the entire image.
